# Evaluation of Withering Quality of Black Tea Based on Multi-Information Fusion Strategy

**DOI:** 10.3390/foods14091442

**Published:** 2025-04-22

**Authors:** Ting An, Yongwen Jiang, Hanting Zou, Xuan Xuan, Jian Zhang, Haibo Yuan

**Affiliations:** 1 National Key Laboratory for Tea Plant Germplasm Innovation and Resource Utilization, Tea Research Institute, Chinese Academy of Agricultural Sciences, Hangzhou 310008, China; anting_mac@163.com (T.A.); jiangyw@tricaas.com (Y.J.); 13500436167@163.com (H.Z.); 2024613022042@stu.zafu.edu.cn (X.X.); 2 School of Intelligent Manufacturing, Huzhou College, Huzhou 313000, China

**Keywords:** MV, NIRS, data fusion strategy, moisture content, black tea withering

## Abstract

The intelligent perception of moisture content (MC) for tea leaves during the black tea withering process is an unsolved task because of the acquisition of limited sample characteristic information. In this study, both the external and internal features of withering samples were simultaneously acquired based on near-infrared spectroscopy (NIRS) and machine vision (MV) technology. Different data fusion strategies, including low-, middle- and high-level strategies, were employed to integrate two types of heterogeneous information. Subsequently, the different fused features were combined with a support vector regression (SVR) algorithm to establish the moisture perception models of withering leaves. The middle-level-variable iterative space shrinkage approach (VISSA) displayed the best performance with 5.7705 for the relative percent deviation (RPD). Therefore, the proposed multi-information fusion strategy could achieve an intelligent perception of tea leaves in the black tea withering process. The integration of NIRS and MV technology overcomes the limitations of single-technology approaches in black tea withering assessment, providing a robust methodology for precision processing and targeted quality control of black tea.

## 1. Introduction

Black tea is one of the world’s renowned beverages, widely favored by consumers for its health benefits and flavor [1]. It is worth noting that the health benefits and flavor of black tea are the results of the combined action of various water-soluble substances, such as catechin, caffeine, soluble sugars, amino acids, and tea pigments. There exists a significant correlation between the substance content and the quality of black tea. As we all know, the manufacturing craft determines the quality of black tea. Remarkably, withering is the first process in the preparation of black tea, and its quality lays the foundation for subsequent manufacturing steps. Generally, the MC is the most critical factor in evaluating the withering quality. The withering leaves with insufficient moisture are prone to damage in the rolling process affecting the appearance quality of the finished tea. Conversely, the excessive moisture of tea leaves could result in the loss of some essential components for finished tea. In actual production, experienced tea makers indirectly assess the withering degree based on the visual and tactile feature changes in tea leaves. This method is susceptible to external factors and cannot accurately obtain the MC of withering leaves. Although the moisture analyzer and gravimetric oven could accurately determine the MC of tea samples, they exhibit significant lag time, rendering them unable to guide practical withering production effectively. Hence, an accurate and rapid non-destructive detection method for MC of withering leaves should be developed.

In recent years, non-destructive testing technology has been widely employed in the assessment of agricultural product quality, particularly during the withering process [2,3,4,5]. Liang et al. obtained the image information of tea samples under different withering durations using MV technology. The color and texture features of these sample images were extracted for the construction of the MC perception model, which demonstrated a satisfactory result with the correlation coefficient of the prediction set (R_p_) for 0.9314 [6]. In a study conducted by our team, the convolutional neural network model based on the confidence levels was proposed for quantitatively predicting the MC values of withering leaves, which demonstrated significant advantages compared with the traditional machine learning strategy [7]. Huang et al. collected tea leaves with different varieties and batches. The MC perception model of tea leaves with different varieties and batches was established based on the Vis-NIR spectroscopy and model transfer strategy [8]. The withering process of black tea is an exceptionally complex procedure involving gradual enzymatic and non-enzymatic reactions. As the samples’ MC values diminish, notable changes occur in both their external appearance and internal quality characteristics. From an external characteristics’ perspective, the color of withering leaves transitions from bright green to dark green, and their texture curls because of the loss of the MC. MV technology achieves a quantitative perception of moisture in withering leaves based on these appearance phenomena [7]. In terms of internal characteristics perspective, as the withering progresses, some significant changes would occur in certain substances, such as the moisture, chlorophyll, and theaflavin content. These alterations in substances would lead to differences in the stretching vibrations of some functional groups at different spectral bands, which is the reason NIRS can assess the MC in withering leaves [9]. Consequently, both MV and NIRS technology are limited to capturing unilateral digital information of withering leaves. Hyperspectral imaging technology (HSI) technology, integrating spectral and image information, could accurately obtain spectral information at the pixel level of samples and form three-dimensional data cubes. An et al. established cross-validation models of the MC for front and back sizes of single withering leaves and produced the moisture distribution map for the local region of withering leaves [10]. In contrast to An’s study, Wei et al. developed the MC prediction model for accumulated withering leaves and created moisture perception maps at different moisture gradients [11]. Remarkably, the MC prediction models constructed by the above methods did not comprise external features of tea leaves. They solely relied on the pixel-level spectral information to produce moisture distribution maps. Hence, obtaining comprehensive samples of digital information and effectively integrating these features are particularly crucial for comprehensively evaluating the withering quality. Wang et al. integrated the visual, olfactory, and gustatory features of tea samples and achieved the qualitative discrimination of withering degree [12]. However, this study did not quantify the sample MC values, which directly affect the withering degree. In a study conducted by Chen et al., the image and spectral features of withering leaves were integrated based on data and feature-level fusion strategy and successfully established the MC perception models of withering leaves [13]. Although these studies have demonstrated the feasibility of assessing the withering quality of black tea based on the low and middle-level fusion strategies, they have not established the MC perception models using decision-level information, nor have they analyzed the contribution of each single information technology to the MC perception model. Hence, developing the MC perception models for withering leaves based on different fusion strategies is a highly meaningful task.

The core principle of data fusion strategies lies in multi-level (low-, mid-, and high-level) integration of heterogeneous information to compensate for the limitations of single techniques. For instance, the NIRS effectively captures the internal chemical compositions of tea leaves yet suffers from limited detection areas and lacks appearance morphological information. Conversely, MV technology monitors macroscopic exterior features across larger areas but cannot obtain internal features. The multi-information fusion strategy enables simultaneous monitoring of both the internal and external features of withering samples, thereby overcoming the limitations of single information technology. Furthermore, this methodology can be extended to acquire and evaluate both internal and external quality parameters of agricultural products, enabling comprehensive quality assessment critical for agricultural products. Hence, the multi-information fusion strategy was employed in this study to evaluate black tea withering quality comprehensively.

In this study, the MV technology was employed to acquire the color and texture features of withering leaves, which were utilized to represent the external visual characteristics of tea leaves. Meanwhile, the NIRS technology was leveraged to capture sensitive bands related to the moisture of withering leaves. Subsequently, the above digital information was integrated by different levels of fusion strategies, leading to the establishment of moisture perception models during the withering process.

## 2. Materials and Methods

### 2.1. Samples

**Figure 1:**
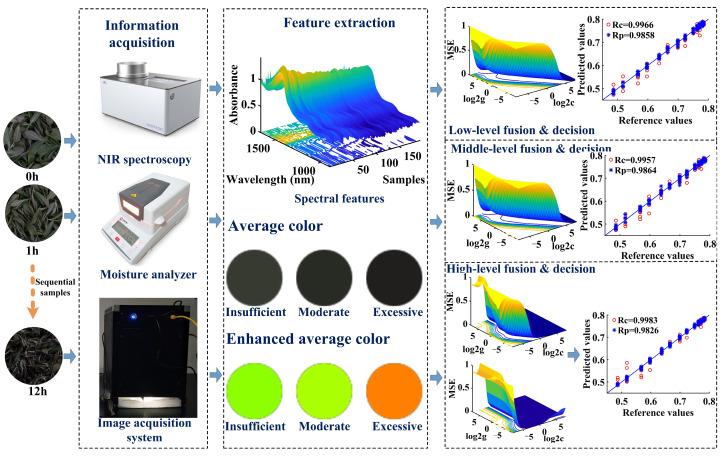
The flow diagram of the withering experiment and data analysis. Note: The closer the color is to yellow, the higher the MSE value; the closer it is to blue, the lower the MSE value.

In this study, 30 kg ‘Jiukeng’ fresh leaves were selected for the black tea withering experiment. These fresh tea leaves were harvested on 14 September 2024 from the experimental tea plantation at the Tea Research Institute of the Chinese Academy of Agricultural Sciences. During collection, the freshly harvested leaves were temporarily stored in a breathable bamboo basket with ice bags to prevent thermal accumulation in accumulated leaves that could compromise quality parameters, then immediately transported to the processing laboratory. Subsequently, the surface condensate water was carefully removed from the fresh tea leaves, and these tea leaves were applied for the withering experiment. The withering experiment lasted for 12 h and encompassed three stages: insufficient, moderate, and excessive withering. Generally, fresh leaves containing more than 62% moisture are classified as insufficient withering. Tea leaves with moisture levels ranging from 58% to 62% indicate moderate withering, while those below 58% are considered excessive withering because of excessive moisture loss [13]. In the withering experiment, these fresh tea leaves were put into a withering trough and placed in a special environment maintained at 3 cm sample thickness, 50% humidity, and 30 °C temperature. These accumulated withering leaves were turned every 1 h to ensure uniform dehydration. To characterize the changes in the withering leaves, several withering leaves were collected every 1 h for the collection of the MC values and digital information of each tea sample using the five-point sampling method. For the MC detection, 3 g tea leaves were placed in the moisture analyzer (MA35M-000230V1, Sartorius, Göttingen, Germany), which demonstrated a readability of 0.01% and a sensor precision of 1 mg, to measure the internal MC of withering samples, and this process was repeated 3 times every hour. The moisture values measured from 3 replicate experiments were averaged to represent the MC values at this specific withering time. Subsequently, the image and spectral information were obtained, respectively. Figure 1 illustrates the process of the withering experiment and relevant data analysis.

### 2.2. Spectra Acquisition by NIRS

The spectral information of withering leaves was collected utilizing a spectrum analyzer (IAS-3100, Intelligent Analysis Service Co., Ltd., Wuxi, China). This spectrum analyzer was constructed based on the dispersion principle of an array MEMS micro-mirror and grating, with a spectral acquisition range covering the near-infrared region of 900–1700 nm. Some parameters, including baseline noise, wavelength repeatability, wavelength accuracy, and absorbance repeatability, were below 0.0001 AU, 0.02 nm, 1 nm, and 0.0002 AU, respectively. Subsequently, several withering leaves (whole leaves) were collected at each withering time using the five-point sampling method. These collected leaves were then evenly divided into 15 samples (approximately 50 g per sample) for the acquisition of the NIR spectrum. Importantly, the spectrums from 5 local regions were acquired for each sample and averaged to represent the spectral features for this specific withering sample. Consequently, a total of 195 spectra of withering samples were obtained, which were subjected to standard normal variate (SNV) for preprocessing. In succession, these withering samples were sent to the MV system for image information collection.

### 2.3. Image Acquisition by Self-Built MV System

The MV system was developed by our research team and consists of these components: an industrial-grade camera, an arc-shaped illumination source, a mounting frame, and a software system. The FI-S200C-G industrial camera (Fenghua Imaging Technology Co., Ltd., Ningbo, China), composed by 4 mm low-distortion lens, 1/2.8″ CMOS sensor, and 1080 × 1080 pixels resolution, was operated with an exposure time of 0.09 ms. The monochromatic white DOME-type arc light source provided uniform illumination at 100 lx intensity throughout the imaging process. A total of 195 images of withering samples were acquired based on a self-built MV system. Subsequently, the 12 color and 6 texture features were extracted from withering sample images, respectively. The 12 color features contained the mean value of the red component (R), green component (G), blue component (B), hue (H), saturation (S), visible light (V), brightness component (L), a component (a), b component (b), super green transformation (2G-R-B), the ratio of the red channel to the green channel (R/G), and the color angle (hab*). The 6 texture features included average gray value (m), standard deviation (δ), smoothness (r), third moment (μ), consistency (U) and entropy (e). These color and texture features were subjected to Normalization for processing.

### 2.4. Data Fusion Strategy

In this study, the spectra and image information were applied to describe the internal and external characteristics of withering leaves, respectively. To comprehensively acquired digital information of withering leaves, a data fusion strategy should be developed. Generally, data fusion strategies are categorized into low (data)-, middle (feature)- and high (decision)-levels [14]. In our research, the spectra and image features of withering leaves were merged into a data matrix, which was regarded as a low-level fusion strategy. For the middle-level fusion strategy, the dimensionality reduction and feature selection were applied to each digital information for the selection of effective variables. The principal component analysis (PCA) is a commonly utilized method for data dimensionality reduction [15]. In contrast to the dimensionality reduction method, the feature selection strategy eliminated irrelevant information from raw data while retaining features that possessed relative independence. In our research, the features selection strategies, including variables combination population analysis and iterative retained information variable algorithm (VCPA-IRIV), the variable iterative space shrinkage approach (VISSA), and competitive adaptive reweighted sampling (CARS) were performed for effective spectra information selection. Meanwhile, the correlation analysis was applied to remove irrelevant color and texture features [10,16,17,18]. The high-level fusion strategy was used to consolidate the decision results from individual digital information to achieve the “ensemble decision”. Multiple linear regression (MLR) was used to integrate the decision information from spectral and image characteristics to explain variations in the moisture of withering leaves in this study. Although the ultimate perception results could be affected by the decision accuracy of each digital information, both NIRS and MV technology can effectively characterize the moisture changes in withering leaves. Hence, the MLR algorithm was applied to integrate the decision results from different digital information:

(1) $$y_{i,ac}=b+k_{1}\times x_{1,\;pc}+k_{2}\times x_{2,pc}+\cdots +k_{n}\times x_{n,pc}$$

(2) $$y_{i,\;pc}=b+k_{1}\times x_{1,pc}+k_{2}\times x_{2,pc}+\cdots +k_{n}\times x_{n,pc}\;$$

(3) $$y_{i,\;pp}=b+k_{1}\times x_{1,pp}+k_{2}\times x_{2,pp}+\cdots +k_{n}\times x_{n,pp}\;$$

The *i* and *n* denote the *i*th sample and *n*th digital information, respectively. The y means the MC values of withering leaves. The *x* represents different digital information. Consequently, the $y_{i,ac}$, $y_{i,\;pc}$ and $y_{i,\;pp}$ demonstrate the actual value of the calibration set, the predicted value of the calibration set, and the predicted value of the prediction set, respectively. The $x_{n,pc}$ and $x_{n,pp}$ denote the predicted value of calibration and prediction set based on the nth digital information, respectively. The Equation (1) was used to determine the coefficient *k* and constant *b*. The Equation (2) was applied to obtain the $y_{i,\;pc}$.

### 2.5. Moisture Perception Model Establishment and Evaluation

In contrast with the support vector machine (SVM) in classification problems, the SVR algorithm attempts to find an “optimal hyperplane” in the feature information [19]. This hyperplane is considered a regression function, aiming to approximate the feature information and predict the targeted outcomes within an acceptable margin of error. Remarkably, the SVR algorithm enhances the robustness of the perception model by maximizing the margin of the regression model. Hence, this algorithm will be applied for the establishment of a withering moisture perception model in this research. In addition, several parameters, including the correlation coefficient of calibration set (R_c_), R_p_, RPD, the root mean square error of the calibration (RMSEC) and prediction (RMSEP) set, were employed to evaluate the performance of the MC perception model for withering leaves.

### 2.6. Software

All data analyses, including feature extraction, multi-information fusion, and predictive modeling, were implemented using MATLAB R2017b (The Math Work, Inc., Natick, MA, USA). The statistical visualizations were created using Origin 2021b (OriginLab Corporation, Northampton, MA, USA).

## 3. Results and Discussion

### 3.1. Digital Information Representation and Analysis of Different Sensors

Some significant changes occurred in both the internal substances and external characteristics of withering samples. To provide a more comprehensive description of the dynamic changes in withering samples, NIRS and MV technologies were utilized to represent the internal and external characteristics of tea leaves digitally. These features from different sensors were represented and analyzed, respectively.

#### 3.1.1. Internal Features of Withering Leaves

**Figure 2:**
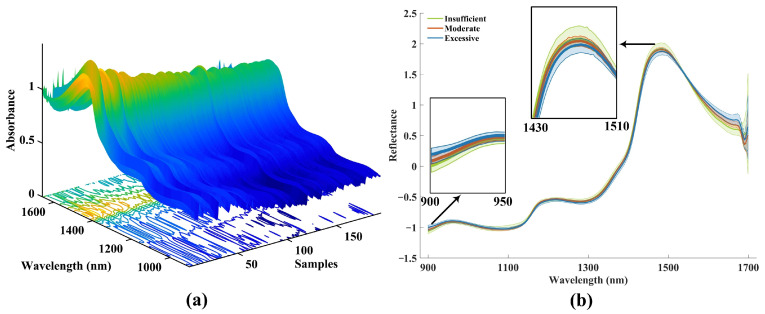
The spectra of withering leaves based on (**a**) raw data and (**b**) the average spectra of different withering degrees based on SNV processing data. Note: The closer the color is to yellow, the higher the absorbance value; the closer it is to blue, the lower the absorbance value.

In this study, the average spectra of each sample were utilized to characterize the internal features of withering leaves. Furthermore, the representative spectra of all withering samples were presented in Figure 2a, which indicated that all the sample spectra exhibited a consistent trend in the range of 900–1700 nm. Although the absorbance of the spectra exhibited a consistent changing trend, there still existed some noise in certain bands that were not readily perceptible to the human eye. Consequently, all spectral data for withering leaves were subjected to the SNV algorithm to eliminate the impact of optical scattering. The average spectra of tea samples with different moisture are displayed in Figure 2b. Obviously, the spectral distribution of all samples becomes more concentrated. After the SNV preprocessing, the spectra of withering samples with different moisture still exhibited the same trend, but there were subtle differences in the spectral profiles. The absorbance near 900 nm and 1650 nm increased over the moisture lost. However, the opposite is true near the absorption peak at 1450 nm. Evidently, these spectral absorbance variations directly correlate with the physical state of withering leaves. As moisture loss occurs, some changes in leaf cell membrane permeability alter light scattering properties, accounting for the observed absorbance increases at specific wavelengths, such as 900 nm and 1650 nm. Notably, the band near 1450 nm represents a strong absorption feature for water corresponding to the O–H first overtone, where higher moisture content yields greater absorbance. The moisture loss during withering directly leads to reduced absorption intensity in this spectral region. Additionally, the spectra of withering leaves also displayed several absorption peaks, such as around 960 nm, 1100 nm, 1170 nm, and 1500 nm. The peaks around 960 nm were attributable to the overtone of OH groups [20]. The peaks near 1100 nm and 1500 nm were related to the second and first overtone of OH groups [21]. The peak near 1170 nm corresponded to the overtone of CH groups [22]. These peaks and their corresponding functional groups for water and catechins demonstrated the feasibility that NIRS technology characterizing the internal features of withering leaves.

#### 3.1.2. External Features of Withering Leaves

**Figure 3:**
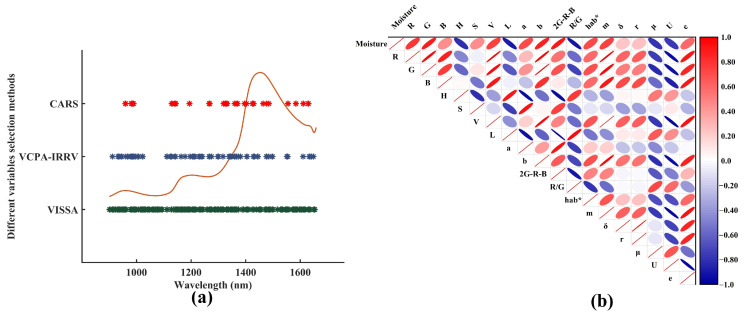
The selected effective features for spectra data (**a**) and image data (**b**).

In actual withering production, the moisture loss progressively concentrates the cell sap, while enzymatic and non-enzymatic reactions drive chlorophyll transformation and theaflavin formation. These biochemical changes induce a visible color transition from bright green to dark green, demonstrating the feasibility of using color-related characteristics, such as basic color, saturation, and brightness components, for assessing withering quality. Furthermore, the moisture loss reduces intercellular space, resulting in increased leaf rigidity, curling morphology, and surface wrinkling. These physical alterations modify light scattering properties, leading to distinct textural changes among samples at different withering times. Consequently, the color and texture information can represent changes in the external features of withering leaves. In this study, the average color and texture features, such as R, G, B, H, S, V, L, a, b, 2G-R-B, R/G, hab* m, δ, r, μ, U, and e, were applied to characterize the external features of withering leaves. Subsequently, a correlation analysis was conducted to characterize the correlations between different variables. As can be seen in Figure 3b, some significant correlations exist among most variables, attributable to the fact that both color and textural features are governed by the same physiological and biochemical processes. The correlations among color features stem from the synergistic effects of the biochemical reactions on pigment composition. For instance, chlorophyll degradation reduces the G value, resulting in coupled variations in derived parameters such as R/G ratio and 2G-R-B. The correlations among texture features are directly associated with physical and structural changes in withering leaves induced by moisture loss. With the loss of moisture, the increasing curling degree of withering samples leads to a synergistic change in texture parameters, evidenced by the significant negative correlation between U and e. The withering of black tea is a complex process, and the correlations among these multidimensional features are a unified manifestation of synergistic interactions between chemical and physical changes during this progression. Therefore, further analysis was required to identify strategies for eliminating redundant information and to assess the contribution of each variable to the moisture perception model.

### 3.2. Effective Feature Selection of Different Digital Information

According to the above description, not all variable features contributed to the construction of the moisture perception model, and some redundant features could degrade the performance of the perception model. Hence, some effective feature selection strategies would be performed. The CARS, VCPA-IRIV, and VISSA algorithms were performed to select effective variables for spectral information, respectively. The Pearson correlation analysis was conducted for the selection of effective color and texture information.

#### 3.2.1. Feature Selection of Spectral Information

After repeated testing, some notable parameters of CARS, VCPA-IRIV, and VISSA algorithms were determined. The sampling runs and cross-validation folds for the CARS algorithm were 50 and 5, respectively. The group number of cross-validation and maximal PCs for the VCPA-IRIV algorithm were 10 and 5, respectively. The maximal PCs and cross-validation folds for the VISSA algorithm were 15 and 5, respectively. The CARS algorithm selected 41 effective features for the moisture perception model of withering leaves. The compression rate of spectral information was 94.6%, indicating that the CARS algorithm considered most of the variables for raw spectral data as redundant information. Compared with the CARS algorithm, the VCPA-IRIV algorithm selected 86 effective features, which was nearly double the number of features chosen by the CARS algorithm. The compression rate of raw spectra was 88.7%. The VISSA algorithm retained 231 effective features for the moisture perception model of withering leaves, and the compression rate of raw spectra only reached 69.6%. These selected variables are displayed in Figure 3a. Obviously, these selected bands were inattentively distributed. However, some effective variables were selected by these three algorithms. The selected bands at around 970 nm were attributable to second stretching overtones for free OH groups. These selected bands near 1200 nm corresponded to second stretching overtones by the absorption of CH_3_ and CH_2_ groups. These selected bands at 1400–1420 nm were related to the first stretching overtones and combination bands of free OH groups. These selected bands are located at 1660–1700 nm due to the first stretching overtones by the absorption of CH_3_ and CH_2_ groups. Although some bands could not represent the overtone of OH, they could represent the overtone of HX, which showed a similar change in the OH groups [23]. Specifically, the selected bands located around 970 nm and 1420 nm, corresponding to the first and second stretching overtones as well as combination bands of OH groups, can directly reflect moisture changes in withering leaves [20,21]. The selected bands near 1200 nm and 1700 nm were related to the catechins content present in withering leaves [22]. Notably, although certain functional groups are closely associated with catechins, both catechins and MC exhibit a significant decreasing trend during withering processing, indicating a strong positive correlation [24]. Consequently, the corresponding spectral bands of these functional groups can directly or indirectly characterize MC changes. This finding provides crucial mechanistic insights for evaluating withering quality using NIRS technology.

#### 3.2.2. Feature Selection of Image Information

The Pearson correlation analysis was performed to assess the degree of response for color and texture variables to the changes in the MC in withering leaves, and the results were displayed in Figure 3b. Of this figure, the *p*-values for these color and texture variables were below 0.01, demonstrating a significant correlation between these variables and the changes in the MC in withering leaves. Obviously, the variables including R, G, B, S, V, a, b, 2G-R-B, hab*, m, δ, r, and e exhibited a positive correlation with the changes in the MC in withering leaves and the correlation coefficients of these variables with respect to changes in the MC were 0.79, 0.89, 0.46, 0.45, 0.76, 0.72, 0.87, 0.90, 0.71 0.76, 0.24, 0.25, and 0.6, respectively. Conversely, the variables, such as H, L, R/G, μ, and U, demonstrated a negative correlation with changes in the MC in withering leaves, and the correlation coefficients of these variables with respect to changes in the MC were −0.80, −0.89, −0.92, −0.78, and −0.77, respectively. In addition, the cutoff line of the correlation coefficient was set to 0.6, indicating that the variables with an absolute correlation coefficient greater than 0.6 were considered to be effective features. Hence, the effective color and texture features, including R, G, H, V, L, a, b, 2G-R-B, R/G, hab*, m, μ, U, and e, were retained for the establishment of moisture perception models.

### 3.3. Moisture Prediction Models with a Single Technology

The moisture perception models for withering leaves were constructed based on the SVR algorithm and each single digital information technology, such as NIRS and MV technologies. Before modeling, the digital information and its corresponding MC values were divided into a calibration set and a prediction set with the radio of 3:1. The performances of established moisture perception models were displayed in Table 1. Remarkably, the R_p_ values of these established models with single digital information were both higher than 0.95, and the RPD values were both higher than 3.5, indicating that both the NIRS and MV technologies demonstrated strong perception capabilities for the moisture of withering leaves. In addition, the established model using NIRS technology displayed better performance than the established model based on MV technology. The color and texture features indirectly reflected the changes in MC in withering leaves. In contrast to MV technology, the NIRS technology provided changes in spectral reflectance, which was directly related to the OH groups for water. Furthermore, each type of digital information could only provide one-sided sample information. Hence, different data fusion strategies were proposed to integrate digital information from different sensors.

### 3.4. Moisture Perception Models with Multi-Level Data Fusion

In this study, the low-, middle- and high-level data fusion strategies were applied to integrate digital information from different sensors. Subsequently, the fused digital information was combined with the SVR algorithm to construct the moisture perception model for withering leaves.

#### 3.4.1. Low-Level Fusion Strategy

The low-level fusion strategy means all digital information is simply concatenated, with the dimension of the integrated information being a composite of the dimensions of each individual piece of digital information. Table 2 displays the prediction results based on this fusion strategy. The R_c_, RMSEC, R_p_, RMSEP, and RPD were 0.9966, 0.0082, 0.9858, 0.0180, and 5.6142, respectively. Obviously, the established moisture perception model based on a low-level fusion strategy displayed better prediction performance than the established model using single pieces of digital information, indicating that acquiring and representing comprehensive information of withering leaves contributed to enhancing the performance of the moisture perception model. However, there existed a certain degree of redundancy among different feature variables, which reduced the running speed and performance of the moisture perception model. Consequently, some features that contributed significantly to the moisture perception model need to be further selected to improve the running speed and performance of the prediction model.

#### 3.4.2. Middle-Level-PCA Fusion Strategy

**Figure 4:**
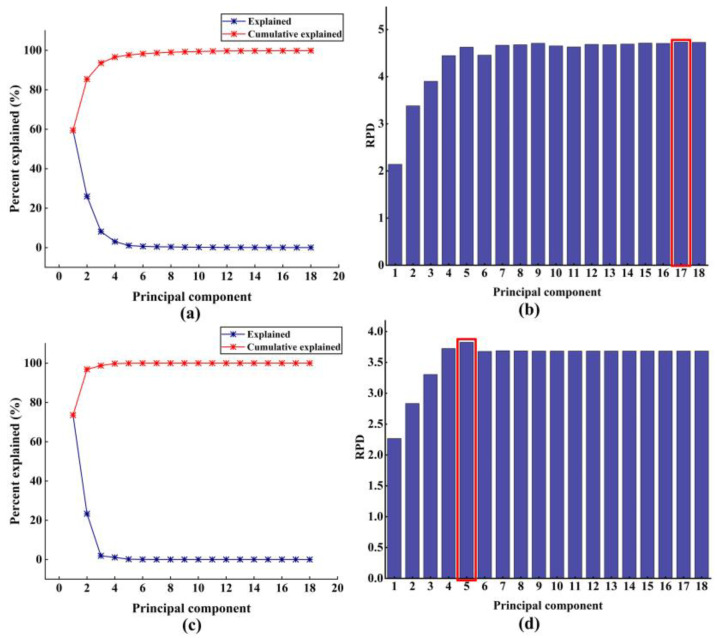
Percent explained and cumulative explained of spectra data (**a**) and image data (**c**) by PCA; SVR prediction models on different numbers of PCs to determine the best RPD value for spectra data (**b**) and image data (**d**).

The middle-level-PCA fusion strategy indicates that the NIRS and MV digital information were subject to the PCA algorithm, respectively. Subsequently, the optimal PCs of each information technology were selected based on the model’s perceptual performance and these selected optimal PCs were concatenated into a feature matrix for the establishment of a moisture perception model. The cumulative explained and the explained percent for the NIRS and MV digital information using the first 18 PCs are demonstrated in Figure 4a,c, respectively. Obviously, the first 18 PCs of NIRS and MV digital information could represent 99.82% and 100% of the information for raw variables, respectively. This phenomenon indicated that the first 18 PCs of NIRS and MV digital information could represent almost all the content of raw sample information. To obtain the optimal PCs of the two types of digital information, the SVR algorithm was employed to establish the moisture perception model, with the results presented in Figure 4b,d. Consequently, the first 17 PCs of raw spectral digital information and the first 5 PCs of image digital information were directly connected to construct the moisture perception model, with the results shown in Table 2. In contrast to each single piece of digital information, the performance of the middle-level-PCA strategy was not satisfactory, indicating that the appropriate data fusion strategy is critical to the predictive performance of the perception model.

#### 3.4.3. Middle-Level-Variable Selection Fusion Strategy

The middle-level-variable selection fusion strategy indicates that the NIRS and MV digital information are subject to different variable selection algorithms and Pearson correlation analysis, respectively. These selected effective features were concatenated into a feature matrix for the establishment of a moisture perception model. For the middle-level-CARS strategy, a total of 55 variables were selected to establish the moisture model, and the compression rate of raw variables was 92.9%. The R_c_, RMSEC, R_p_, RMSEP, and RPD were 0.9922, 0.0125, 0.9854, 0.0193, and 5.2756, respectively. For the middle-level-VCPA-IRIV, 100 variables were retained to establish the moisture perception model for withering leaves, and the compression rate of raw variables was 87.1%. The R_c_, RMSEC, R_p_, RMSEP, and RPD were 0.9932, 0.0116, 0.9859, 0.0188, and 5.4320, respectively. For the middle-level-VISSA strategy, 245 variables were selected for the establishment of the moisture perception model, and the compression rate of raw variables was 68.5%. The R_c_, RMSEC, R_p_, RMSEP, and RPD were 0.9957, 0.0092, 0.9864, 0.0178, and 5.7705. The results are displayed in Table 2. Obviously, as the number of variables decreases, the performance of the perception model decreases, indicating that some important bands for spectral information were removed.

#### 3.4.4. High-Level Fusion Strategy

The high-level fusion strategy means that the decision results for each single piece of digital information are fused to construct the moisture perception model. The multiple linear fitting equations are displayed using Equation (4). Based on the weight coefficients of the spectra and image information in the linear regression equation of the MC, the order of two sensors to the improvement of model performance was NIRS > MV. The same conclusion was obtained by the modeling results of each single digital information (displayed in Table 1). The values of R_p_, RMSEP, and RPD for the moisture perception model using a high-level fusion strategy were 0.9826, 0.0186, and 5.3456, respectively. The high-level fusion strategy demonstrated satisfactory predictive performance, primarily attributed to the reliable decision outcomes provided by each single digital information.

The established MC perception model uses a high-level fusion strategy.

(4) $$y=0.60339x_{1}\;+\;0.42081x_{2}\;-\;0.01610$$

The parameters, including $x_{1}$, $x_{2}$, and y, mean the predicted value of NIRS, MV, and the actual value of moisture content, respectively.

### 3.5. Model Comparison and Evaluation

The performance of the moisture perception model based on each single piece of digital information and the fusion digital information were proposed and compared. Obviously, most perception models using data fusion strategies performed better predictive performance than the established models based on single digital information, except for the middle-level fusion-PCA strategy. The results demonstrated that not all data fusion strategies could enhance the performance of the perception model. Although the obtained PCs using the PCA method could represent over 99% of raw data information, not all PCs exhibited specific responses to the research target, resulting in the unsatisfactory predictive performance of the perception model. In contrast to the middle-level-PCA strategy, the middle-level-VISSA strategy was considered the best moisture perception model for withering leaves because it showed the best RPD value. For the color and texture features, 14 variables, including R, G, H, V, L, a, b, 2G-R-B, R/G, hab*, m, μ, U, and e were selected. For these spectral data, 231 effective bands were retained, and they might be related to the second overtone of OH and CH [21,22]. During the withering process of black tea, the moisture loss was very obvious and also accompanied by the enzymatic reaction and reduction of compounds containing hydrogen. Therefore, some other important components, such as catechins, amino acids, and soluble sugars, changed significantly [25,26]. There is a certain correlation between the changes in these substances and the moisture. These effective features selected by the middle-level-VISSA strategy could directly or indirectly represent the changes in moisture in withering leaves. Hence, they could be applied for the evaluation basis for the MC in withering leaves.

Liang et al. and Zhang et al. constructed the moisture perception model of tea leaves based on the digital information for MV and NIRS, respectively [6,27]. Their moisture perception models based on single digital information achieved satisfactory results. However, our proposed moisture perception model exhibited better predictive performance, demonstrating that the fusion of comprehensive feature information of tea samples is indeed beneficial for improving the performance of prediction models. In addition, Chen et al. established the moisture prediction model of withering leaves based on low- and middle-level fusion strategies [13]. They believed that the middle-level-RF (cut of line = 0.8) displayed the best predictive performance, with 5.5596 for RPD. In contrast to Chen’s study, our research proposed the high-level fusion strategy to further characterize the contribution of each single piece of digital information to the moisture perception model. In addition, our proposed middle-level-VISSA strategy displayed a higher RPD value, indicating that our constructed moisture perception model has better predictive performance.

In this study, the proposed data fusion strategy effectively overcomes the limitations of individual techniques, including restricted detection range and penetration depth, by integrating complementary information from NIRS (internal features) and MV (external features). Our proposed methods enable comprehensive characterization of withering samples, thereby facilitating the development of more accurate quality assessment models. This strategy significantly enhances the precision of withering quality control in actual production.

## 4. Conclusions

(1) This study proved the feasibility of the perception of the MC values in withering leaves based on the NIRS combined with MV technology. The multi-information fusion strategy overcomes the limitations of single digital information that can only capture partial feature information, providing a novel methodology for the comprehensive characterization of tea samples.
(2) NIRS technology demonstrates superior moisture detection capability compared with MV technology. This discovery provides valuable insights into refining fusion information models and developing specialized equipment in subsequent research.
(3) An appropriate data fusion strategy could remove redundant features and improve the accuracy of the moisture prediction model for withering leaves. The middle-level-VISSA strategy achieves the most accurate moisture perception. The selection of multi-information fusion strategies is critically important for accurate perception and precise control of moisture content during withering processing.

## Figures and Tables

**Table 1:** SVR models for moisture content of withering leaves using individual data.

Data	No. of Variables	Parameter		Calibration Sets		Prediction Sets		
		*c*	*g*	R_c_	RMSEC	R_p_	RMSEP	RPD
NIR	760	0.4353	1.3195	0.9971	0.0078	0.9775	0.0210	4.7258
MV	18	0.4353	0.0206	0.9958	0.0094	0.9657	0.0260	3.6824

**Table 2:** SVR models for moisture content of withering leaves using multi-level data fusion.

Level	Methods	No. of Variables	Parameter		Calibration Sets		Prediction Sets		
			*c*	*g*	R_c_	RMSEC	R_p_	RMSEP	RPD
Low-level		778	3.0314	256	0.9966	0.0082	0.9858	0.0180	5.6142
Middle-level	PCA	22	0.7579	0.0156	0.9964	0.0086	0.9669	0.0260	3.7149
	CARS	55	0.5743	256	0.9922	0.0125	0.9854	0.0193	5.2756
	VCPA-IRIV	100	1	256	0.9932	0.0116	0.9859	0.0188	5.4320
	VISSA	245	5.2780	256	0.9957	0.0092	0.9864	0.0178	5.7705
High-level	MLR				0.9883	0.0057	0.9826	0.0186	5.3456

## Data Availability

The original contributions presented in this study are included in the article. Further inquiries can be directed to the corresponding authors.
